# The Relationship Between Serum Neurosteroids and Oxytocin Levels and Craving, Aggression and Emotion Regulation in Patients with Methamphetamine Use Disorder

**DOI:** 10.3390/medsci14020169

**Published:** 2026-03-27

**Authors:** Hacer Akbas Cakmak, Ahmet Bulent Yazici, Derya Guzel Erdogan, Yavuz Selim Ogur, Esra Yazici

**Affiliations:** 1 Department of Psychiatry, Sakarya Training and Research Hospital, Sakarya 54100, Türkiye; hacerakbas9@gmail.com; 2 Department of Psychiatry, Faculty of Medicine, Sakarya University, Sakarya 54290, Türkiyeeyazici@sakarya.edu.tr (E.Y.); 3 Department of Physiology, Faculty of Medicine, Sakarya University, Sakarya 54290, Türkiye

**Keywords:** attachment, emotion regulation, aggression, methamphetamine use disorder, neuroactive steroids, oxytocin

## Abstract

**Background**: This study aimed to investigate the relationship between serum neuroactive steroids (NAS) and oxytocin and craving and psychosocial functioning in men diagnosed with methamphetamine use disorder (MUD). **Methods**: In this observational cross-sectional study, 40 men with MUD (PG) and 41 non-substance-use-disorder controls (CG) completed measures of emotion dysregulation (DERS-16), attachment (ECR-R), aggression (BPAQ), and suicidal ideation (BSS). PG additionally completed the Substance Craving Scale (SCS) and Addiction Profile Index (API). Serum allopregnanolone (ALLO), DHEAS, testosterone, 17β-estradiol (E2), and oxytocin were assayed. **Results**: The results indicated that the PG exhibited significantly higher scores than the CG across all psychological measures. Robust adjusted group effects were observed for DERS-16 (Model 1: F = 35.507, *p* < 0.001; Model 2: F = 18.225, *p* < 0.001) and trait anger (Model 1: F = 41.104, *p* < 0.001; Model 2: F = 16.732, *p* < 0.001). Notably, serum levels of ALLO, DHEAS, testosterone, E2, and oxytocin did not differ significantly between groups. However, hormonal measures were strongly intercorrelated within both groups (r ≈ 0.877–0.936, all *p* < 0.001). In the PG, craving demonstrated positive correlations with DHEAS (r = 0.384, *p* = 0.014), testosterone (r = 0.415, *p* = 0.008), E2 (r = 0.360, *p* = 0.023), and oxytocin (r = 0.350, *p* = 0.027). A multivariable model analyzing craving was statistically significant (R^2^ = 0.350; F(3,36) = 6.474, *p* = 0.001), with composite hormonal factor (B = 2.390, *p* = 0.016) serving as an independent predictor, while API Excluding Craving(API-EC) (*p* = 0.094) and DERS-16 did not emerge as a significant factor (*p* = 0.056). In hormone-specific models controlling for API-EC and DERS-16, DHEAS (*p* = 0.012), testosterone (*p* = 0.007), oxytocin (*p* = 0.023), and E2 (*p* = 0.023) retained significance after false discovery rate (FDR) correction; ALLO did not (*p* = 0.055). **Conclusions**: Despite the absence of significant differences in peripheral NAS and oxytocin levels between groups, men with MUD exhibited pronounced psychosocial impairments. The craving experienced during inpatient treatment was primarily elucidated by an integrated endocrine profile. These findings underscore the necessity for larger longitudinal studies incorporating repeated hormonal assessments to further explore these relationships.

## 1. Introduction

Psychoactive substance use is an important social, economic, and health problem that is becoming increasingly common worldwide [1]. In recent years, methamphetamine use has become an increasingly prominent contributor to the global burden of psychoactive substance use, given its strong associations with severe mental health harms—including psychosis, suicidality, and violence [2,3,4]. It was reported that 1.6 million adults in the USA used methamphetamine each year between 2015 and 2018 [5]. According to the 2022 European Union Drugs Agency (EUDA) report, amphetamine and methamphetamine users constituted at least 15 percent of individuals initiating treatment for substance use disorder (SUD) in Europe [6].

Methamphetamine, first synthesized from ephedrine by Akira Ogata in 1919, evolved from a widely prescribed medical stimulant and military performance-enhancing agent during World War II and later wars into a major substance of abuse with expanding illicit production [7]. Although methamphetamine may be used to enhance attention, wakefulness, and perceived mental performance, the available evidence suggests that such effects are modest, short-lived, and dose dependent. Moreover, these limited effects are clearly outweighed by its substantial risks, including addiction, psychosis, cognitive impairment, and broader psychiatric and medical harms [8,9,10]. Beyond these well-established harms, evidence suggests that substance use can exacerbate difficulties in emotion regulation in the long term due to changes in the prefrontal cortex and amygdala [11,12]. Moreover, ineffective emotion regulation is considered a potential maintaining factor in SUD and may contribute to craving behavior [11,13,14].

Problems with attachment and emotion regulation have also been associated with alcohol, substance, and even behavioral addictions [14]. Studies examining the relationship between attachment and substance use have shown that individuals with secure attachment patterns exhibit lower substance use, while individuals with insecure attachment patterns demonstrate higher substance use [15,16,17].

The term “neuroactive steroids (NAS)” refers to cholesterol metabolites including steroids synthesized in the adrenal glands, gonads, or partially metabolized in the brain, which interact with neural circuits [18,19]. NAS include pregnenolone, pregnenolone sulfate, progesterone, allopregnanolone (ALLO), dehydroepiandrosterone (DHEA), dehydroepiandrosterone sulfate (DHEAS), testosterone, and estradiol (E2). These steroids exert their effects through interactions with presynaptic receptors and ion channels, such as sigma-1 receptors, α1 receptors, nicotinic receptors, D1 dopamine receptors, NMDA receptors, and GABA-A receptors [20]. Notably, human and animal studies show that alcohol, benzodiazepines, and ALLO have a dose-dependent, biphasic effect on behavior and emotion regulation via GABA-A receptors [21]. In addition, the other receptors and receptor systems that NAS interact with are targets for both etiological research and therapeutic interventions in many disorders, including addiction-related conditions [22]. Similarly, oxytocin plays an important role in establishing social bonds, regulating stress, and supporting mental health, but its effects are largely dependent on contextual and individual factors such as attachment style, trauma history, and social environment [23]. Emerging evidence also suggests that oxytocin may attenuate addiction-related behaviors by modulating glutamatergic neurotransmission within the brain’s reward circuitry, thereby counteracting drug-induced neurochemical alterations [24].

Despite these insights, research on the etiology of emotion dysregulation in individuals with methamphetamine use disorder (MUD) remains limited, and further investigation from diverse perspectives is warranted. Although the relationship between oxytocin and attachment as well as emotion regulation is well established, the interaction between oxytocin, emotion regulation, and bonding in substance use disorders remains relatively unexplored and thus merits further research [23,25,26]. Given the multifactorial nature of addiction and addiction-related mental health problems, it is essential to examine multiple interacting factors, such as neuroactive steroids and oxytocin, and their impact on addictive processes like craving, to better understand emotion regulation difficulties in individuals with MUD [27].

Although a limited number of preclinical and clinical studies have investigated the relationship between NAS and SUD, the existing evidence remains insufficient. To the best of our knowledge, no previous study has simultaneously examined emotion regulation and attachment in SUD patients along with NAS and oxytocin. Our hypotheses are as follows: Compared with controls, individuals with methamphetamine use disorder (MUD) are expected to show higher levels of emotion dysregulation, insecure attachment, aggression, and suicidal ideation. We further hypothesize that serum levels of neuroactive steroid steroids (e.g., allopregnanolone, DHEAS, testosterone, estradiol) and oxytocin will differ between the MUD and control groups. Within the MUD group, endocrine indices may be associated not only with craving severity but also with emotion dysregulation and aggression, and psychological and endocrine variables, when considered jointly, may explain meaningful variance in these clinical outcomes.

## 2. Materials and Methods

This study was conducted as an observational cross-sectional study with non-substance use disorder controls. It was carried out at Sakarya Training and Research Hospital (STRH), at the Inpatient Detoxification Centre (IDC) for addiction, between 1 March 2022 and 15 September 2022. Since studies have indicated that different addictive substances may have varying effects on NAS and oxytocin levels [28,29,30,31,32,33], only patients diagnosed with MUD were included in the study to reduce the confounding effects of other substances on serum NAS hormone levels. Female patients were not included to avoid potential hormonal fluctuations associated with the menstrual cycle, which might influence serum NAS levels [25,26,27]. Male patients diagnosed with stimulant use disorder according to DSM-5, who had been using methamphetamine regularly for at least 1 year, and who met inclusion and exclusion criteria, were included. Methamphetamine use was verified by urine toxicology tests, and patients were enrolled after their urine tested negative for methamphetamine metabolites. Controls without substance use disorder, matched to the patient group in terms of gender, age, and living in the same region, were clinically evaluated according to pre-defined exclusion criteria and included as the control group.

### 2.1. Sample Selection

Sample size was estimated using G*Power 3.1.9.7 based on the large between-group difference in serum oxytocin reported previously (Cohen’s d = 0.984) [34]. With α = 0.05 and 95% power, the required sample size was 24 participants per group (total *N* = 48; actual power ≈ 95.7%, one-tailed). Therefore, our planned enrolment of 40 patients and 40 controls was expected to provide very high power (≈99%) for detecting a similar effect. Group differences and effect sizes will be reported with 95% confidence intervals; correlation analyses were considered exploratory. Of the 190 patients admitted to the STRH-IDC between 1 March 2022 and 15 September 2022, 13 were women, and 14 were admitted to the clinic for retreatment during the study period. Of the remaining 177 male patients, 50 were diagnosed with alcohol use disorder, 17 with opioid use disorder, 10 with marijuana and synthetic cannabinoid use disorder, 37 with multiple substance use disorder, 3 with benzodiazepine and pregabalin use disorder, 2 with cocaine use disorder, and 1 with pathological gambling disorder. Of the 57 patients diagnosed with methamphetamine use disorder (MUD), 9 were excluded due to ongoing psychotic symptoms, 1 due to HIV positivity, and 1 due to additional medical diseases and a high body mass index (BMI ≥ 30 kg/m^2^). Additionally, six patients were discharged early before achieving negative urine test results for methamphetamine metabolites, which was an inclusion criterion for the study. Forty patients who met the inclusion criteria consented to participate and signed the informed consent form were ultimately included.

The inclusion criteria for patients were as follows: volunteering for the study, being aged between 18 and 65 years, having a diagnosis of MUD according to DSM-5 criteria, absence of other substance or alcohol use disorders (except nicotine use disorder and cannabis [THC] positivity), and no additional medical conditions, endocrine disorders, or hormone-related diseases. Participants in the MUD group were explicitly required not to meet criteria for other substance use disorders to ensure that observed effects on neuroactive steroid and oxytocin levels were specifically associated with methamphetamine use, thus minimizing the confounding influence of other substances.

The control group consisted of 41 volunteers without substance use disorder (non-SUD controls), recruited from hospital staff and members of the local community who responded to announcements at Sakarya Training and Research Hospital (STRH). Participants in the control group were matched to the patient group based on age, gender, and region of residence. Clinical evaluations using the Structured Clinical Interview for DSM-5 Disorders, Clinician Version (SCID-5-CV), and personality disorders were assessed using the Structured Clinical Interview for DSM-5 Personality Disorders (SCID-5-PD) [35,36]. Inclusion criteria for the control group were as follows: volunteering to participate in the study, aged between 18 and 65 years, absence of any substance use disorder other than nicotine use disorder according to DSM-5 criteria, no alcohol, benzodiazepine, or caffeine dependence, absence of additional medical conditions, endocrine disorders, or hormone-related diseases, having sufficient mental capacity to participate in the study, and being literate. Written informed consent was obtained from all participants.

### 2.2. Sociodemographic and Clinical Measures

#### 2.2.1. Sociodemographic Data Form

The Sociodemographic Data Form is a structured interview form that includes demographic and clinical information, such as age, marital status, education level, occupation, place of residence, monthly income level, migration history, age of starting smoking, age of first substance use, previous treatment history, and presence of withdrawal symptoms.

#### 2.2.2. Addiction Profile Index (API)

The Addiction Profile Index (API) is a self-report scale consisting of 37 questions. It is scored on a five-point Likert-type scale, ranging from 0 to 4. The API evaluates the severity of alcohol and substance addiction, the impact of addiction on the person’s life, motivation for treatment, and substance craving. We also calculated an API-EC score, defined as the Addiction Profile Index total score excluding the craving subscale, to reduce the risk of high correlation with the SCS score [37].

#### 2.2.3. Difficulties in Emotion Regulation Scale-16 (DERS-16)

The Difficulties in Emotion Regulation Scale-16 (DERS-16) consists of 16 items covering 5 subscales [38]. Items on the DERS-16 are rated on a 5-point Likert scale. Higher scores indicate greater difficulties in emotional regulation.

#### 2.2.4. Experiences in Close Relationships Inventory (ECR-R)

The Experiences in Close Relationships–Revised (ECR-R) is a published 36-item self-report questionnaire assessing adult attachment in romantic relationships, with two 18-item subscales measuring attachment anxiety and attachment avoidance. The ECR-R is a revised version of the original ECR, and its items were selected using item response theory methods. Participants rate items on a 7-point Likert scale (1 = strongly disagree to 7 = strongly agree) [39,40].

#### 2.2.5. Buss–Perry Aggression Questionnaire (BPAQ)

The Buss–Perry Aggression Questionnaire (BPAQ) is a Likert-type scale consisting of 29 items designed to measure trait aggression, encompassing dimensions of physical aggression, verbal aggression, hostility, and trait anger [41].

#### 2.2.6. Substance Craving Scale (SCS)

The Substance Craving Scale (SCS) is a self-report scale designed to measure substance craving. It consists of five items, each rated from 0 to 6 points, with a maximum total craving score of 30 [42].

#### 2.2.7. Beck Scale for Suicide Ideation (BSS)

The Beck Scale for Suicide Ideation (BSS) is a clinician-administered scale consisting of 19 items across five screening areas. Each item is scored between 0 and 2, and the total score ranges from 0 to 48. There is no specific cutoff score to classify severity or guide patient management; instead, higher scores reflect an increased risk of suicide [43].

### 2.3. Initial Interview with Participants and Measurements

Patients who were hospitalized for treatment at STRH-IDC and met the inclusion criteria were informed about the study, and their written informed consent was obtained. Participants underwent clinical assessments using SCID-5-CV and SCID-5-PD. Participants also completed a sociodemographic data form to collect demographic and clinical information. Clinical interviews, the sociodemographic data form, psychometric assessments, and blood sample collection were carried out by the same researcher, whereas laboratory analyses were performed separately by a different researcher. Blood samples were collected at 8:00 AM (after a 12 h overnight fast) to determine serum levels of ALLO, DHEAS, testosterone, 17 beta-estradiol (E2), and oxytocin from patients who had completed their first week of hospitalization and whose urine tested negative for methamphetamine metabolites. Blood samples were also taken from the control group at 8:00 AM, following a 12 h overnight fast, to measure serum oxytocin and neuroactive steroid levels. Prior to conducting blood sampling, SCS was administered only to the patient group, whereas the ECR-R, DERS-16, BPAQ, and BSS were administered to both the patient and control groups.

### 2.4. Blood Samples

A 3 mL venous blood sample was collected from each participant in both the patient and control groups into anticoagulant-free tubes between 8:00 and 8:30 AM, following a 12 h overnight fast. Samples were left at room temperature for 2 h to allow coagulation. Subsequently, samples were centrifuged at 1000× *g* for 15 min at 4 °C. The resulting serum was transferred into Eppendorf tubes and stored at −80 °C. After all samples had been collected, they were thawed once, brought to room temperature, and analyzed in a single assay session. Serum levels of allopregnanolone (Catalog No: 201-12-0989, SunRed, Wuxi, China), DHEAS (Catalog No: 201-12-1058, SunRed, Wuxi, China), testosterone (Catalog No: 201-12-1037, SunRed, Wuxi, China), 17 beta-estradiol (Catalog No: E3783Hu, Bioassay Technology Lab, Shanghai, China), and oxytocin (Catalog No: 201-12-1047, SunRed, Wuxi, China) were measured using commercial enzyme-linked immunosorbent assay (ELISA) kits according to the manufacturer’s instructions. The absorbance was measured at a wavelength of 450 nm. According to the kit documentation, the assay sensitivity and working ranges were 0.174 ng/mL and 0.2–60 ng/mL for allopregnanolone, 0.285 nmol/mL and 0.3–50 nmol/mL for DHEAS, 0.086 ng/mL and 0.1–30 ng/mL for testosterone, 7.78 ng/L and 15–3000 ng/L for 17 beta-estradiol, and 1.775 pg/mL and 2–600 pg/mL for oxytocin. The reported intra-assay/inter-assay coefficients of variation were <10%/<12% for allopregnanolone, DHEAS, testosterone, and oxytocin, and <8%/<10% for 17 beta-estradiol. Serum concentrations of allopregnanolone and testosterone were expressed in ng/mL, 17 beta-estradiol in ng/L, DHEAS in nmol/mL, and oxytocin in pg/mL.

### 2.5. Statistical Analysis

The study data were entered into the SPSS 22.00 program on a computer running the Windows 11.0 software package and evaluated using this program. Statistical analyses were conducted to examine group differences and predictive relationships among study variables. The normality of continuous variables was assessed using skewness and kurtosis values [44]. Between-group comparisons were performed for continuous outcomes and categorical outcomes using standard parametric or non-parametric procedures for two independent groups, depending on distributional assumptions and expected cell counts. Bivariate associations were evaluated using Pearson correlation analyses in the full sample and within each group; the subset with additional outcome data was analysed separately where applicable.

For group-dependent associations, general linear models (univariate ANCOVA; Type III sums of squares) were fitted including group membership, a continuous predictor, and their interaction, with covariate adjustment implemented in two stages (a basic model including key demographic/clinical covariates and an extended model additionally including psychosocial covariates). When the homogeneity of variance assumption was violated, findings were corroborated using hierarchical linear regression with bias-corrected and accelerated bootstrapping (5000 resamples).

For the continuous outcome assessed in the subsample, multiple linear regression (Enter method) was used. To derive the hormone composite index, a principal component analysis (PCA) was conducted on the log-transformed hormone variables. To characterize biomarker-specific effects while reducing multicollinearity, additional models were estimated in which biomarkers were entered one at a time after log transformation. Model assumptions were evaluated using collinearity diagnostics (VIF) and the Durbin–Watson statistic. To account for multiple testing across biomarker-specific models, *p* values were adjusted using the Benjamini–Hochberg false discovery rate procedure. All tests were two-tailed with α = 0.05 and 0.95 CI.

## 3. Results

### 3.1. Comparison of Sociodemographic and Clinical Characteristics of Patient and Control Groups

Forty men with MUD and forty-one male controls were included in the study. Sociodemographic and clinical characteristics of the study participants are presented in Table 1. No statistically significant difference was found between the patient and control groups in terms of age, place of residence, duration of breastfeeding in infancy, and migration history. However, significant differences were observed in marital status, educational status and employment status.

Regarding SCID-5-CV diagnoses, no significant differences were found except for nicotine use disorder and MUD. SCID-5-PD diagnoses and a history of suicide attempts were significantly more frequent in the patient group compared to controls. Additionally, the age of smoking initiation and body mass index (BMI) were significantly lower in the patient group.

### 3.2. Comparison of Hormone Levels and Clinical Scales

The patient group had significantly higher scores on the DERS-16, ECR-R, BPAQ, and BSS scales compared to the control group (Table 2).

There was no significant difference in serum levels of ALLO, DHEAS, testosterone, 17 beta-estradiol (E2), and oxytocin between the two groups.

### 3.3. Correlation Analyses in Total Sample

In the full sample (N = 81), scale total scores were positively intercorrelated. DERS-16 Total showed the strongest association with BPAQ Total (r = 0.668, *p* < 0.001), and moderate correlations with ECR-R Anxiety (r = 0.547, *p* < 0.001), ECR-R Avoidance (r = 0.408, *p* < 0.001), and BSS Total (r = 0.311, *p* = 0.005). BPAQ Total was also moderately correlated with ECR-R Anxiety (r = 0.504, *p* < 0.001) and ECR-R Avoidance (r = 0.425, *p* < 0.001) and was positively correlated with BSS Total (r = 0.350, *p* = 0.001). ECR-R Anxiety and ECR-R Avoidance were moderately correlated (r = 0.464, *p* < 0.001), and each showed small positive correlations with BSS Total (r = 0.219, *p* = 0.049; r = 0.243, *p* = 0.029, respectively).

At the subscale level, emotion regulation difficulties were broadly associated with higher aggression and higher insecure attachment. Within aggression facets, hostility and anger showed the most consistent links with insecure attachment and suicide ideation, whereas physical aggression was comparatively less related to suicide ideation (see Supplement Table S1).

In the full sample (N = 81), the only statistically significant association between the sociodemographic variables and the main scale total scores was a negative correlation between BMI and ECR-R Anxiety (r = −0.243, *p* = 0.029). All other correlations between age, breastfeeding duration (months), and BMI with the remaining scale scores (DERS-16 Total and subscales, BPAQ Total and subscales, ECR-R Avoidance, and BSS) were not significant (*p* > 0.05). (see Supplement Table S2). Across the full sample, hormonal measures were highly intercorrelated (approximately r = 0.877–0.936, all *p*s < 0.001) (Supplement Table S3).

In the full sample (N = 81), DHEAS showed small negative correlations with BPAQ Anger (r = −0.258, *p* = 0.020) and DERS Clarity (r = −0.221, *p* = 0.047). All other correlations between the hormone variables and age, BMI, smoking initiation age, DERS total and other DERS subscales, BPAQ total and other BPAQ subscales, ECR-R Anxiety, ECR-R Avoidance, and BSS total were not significant (*p* > 0.05) (see Supplement Table S4).

### 3.4. Correlation Analyses in Patient Group

In the patient subsample (*n* = 40), craving was positively correlated with DERS-16 (r = 0.359, *p* = 0.023) and with DHEAS (r = 0.384, *p* = 0.014), testosterone (r = 0.415, *p* = 0.008), E2 (r = 0.360, *p* = 0.023), and oxytocin (r = 0.350, *p* = 0.027), whereas API total was positively correlated with DERS-16 (r = 0.409, *p* = 0.009) and API-EC score with craving (r = 0.367 *p* = 0.020). DERS-16 correlated positively with BPAQ (r = 0.549, *p* < 0.001) and ECR-R anxious (r = 0.434, *p* = 0.005). Age was negatively correlated with each hormone in patients (DHEAS: r = −0.467, *p* = 0.002; testosterone: r = −0.438, *p* = 0.005; ALLO: r = −0.523, *p* = 0.001; E2: r = −0.470, *p* = 0.002; oxytocin: r = −0.493, *p* = 0.001). In the patient group, hormones were highly intercorrelated, consistent with the pattern observed in the total sample, and these coefficients are provided in the Supplementary Materials. In addition, all other non-significant correlations examined in the patient group are also reported in Supplement Table S5.

### 3.5. Correlation Analyses in the Control Group

In the control group (*n* = 41), hormones were strongly intercorrelated (r = 0.841–0.933, all *p* < 0.001). ALLO (r = −0.320, *p* = 0.042) and testosterone (r = −0.310, *p* = 0.048) were negatively correlated with DERS-16 total, whereas other hormone–DERS associations were not significant (all *p* > 0.05). DERS-16 total was positively correlated with BPAQ total (r = 0.348, *p* = 0.026), ECR-R avoidant (r = 0.599, *p* < 0.001), and ECR-R anxious (r = 0.474, *p* = 0.002). BPAQ total was positively correlated with ECR-R avoidant (r = 0.481, *p* = 0.001) and ECR-R anxious (r = 0.494, *p* = 0.001). The full correlation matrix is provided in Supplement Table S6.

### 3.6. Hormones and Emotion Regulation (DERS-16): ANCOVA Moderation Models

To test whether hormone–DERS-16 associations differed between patients and controls, we fitted GLM univariate ANCOVA models (Type III SS) including Group, each mean-centered hormone (ALLO or testosterone), and the Group × hormone interaction. Because homogeneity of variance was violated (significant Levene tests), models were additionally checked using hierarchical linear regression with BCa bootstrapping (5000 resamples). For ALLO, the Group × ALLO interaction was not significant in Model 1 (age, BMI, education), F = 0.647, *p* = 0.424, or Model 2 (additionally ECR-R anxious, ECR-R avoidant, and BSS), F = 0.795, *p* = 0.455. The group effect was significant in both models (Model 1: F = 35.507, *p* < 0.001; Model 2: F = 18.225, *p* < 0.001), with higher adjusted DERS-16 scores in patients than controls (Model 1 adjusted means: 44.438 vs. 28.462; Model 2: 41.824 vs. 31.056). Parallel results were observed for testosterone: the Group × testosterone interaction was not significant in Model 1 (F = 0.858, *p* = 0.357) or Model 2 (F = 0.408, *p* = 0.525), while the group effect remained significant (Model 1: F = 35.770, *p* < 0.001; Model 2: F = 18.237, *p* < 0.001). In Model 2, ECR-R anxious was significant (ALLO model: F = 12.152, *p* = 0.001; testosterone model: F = 12.024, *p* = 0.001), whereas ECR-R avoidant (*p*s = 0.412–0.413) and BSS (*p*s = 0.281–0.290) were not.

### 3.7. Trait Anger and Testosterone

ANCOVA models examining Buss–Perry Anger indicated heteroscedasticity (Levene’s *p*s = 0.029 and 0.003 for Models 1 and 2). Covariate sets were identical to those used in the DERS-16 models; only the dependent variable differed (Buss–Perry Anger vs. DERS-16). The group × testosterone interaction was not significant in Model 1 (F = 1.547, *p* = 0.218) or Model 2 (F = 0.605, *p* = 0.439). However, the group effect was robust (Model 1: F = 41.104, *p* < 0.001, R^2^ = 0.383; Model 2: F = 16.732, *p* < 0.001, R^2^ = 0.434), with higher adjusted anger scores in patients than controls (Model 1: 16.369 vs. 7.366; Model 2: 14.787 vs. 8.953). Testosterone showed a trend-level main effect in Model 1 (F = 3.587, *p* = 0.062) and a significant main effect in Model 2 (F = 4.604, *p* = 0.035). In bootstrap-confirmed regression, testosterone was negatively associated with anger in the fully adjusted model (B = −5.788, *p* = 0.031; bootstrap *p* = 0.006; BCa 0.95 CI [−9.940, −2.153]), whereas the interaction term remained non-significant.

### 3.8. Craving: Regression Models

A multiple linear regression (Enter method) was conducted with craving as the dependent variable (*n* = 40), entering API-EC, DERS-16, and a hormone composite index. entered as predictors. The hormone composite index was derived using principal component analysis (PCA) based on the hormone variables (including ALLO, DHEAS, testosterone, and E2). Sampling adequacy was good (KMO = 0.857), and Bartlett’s test of sphericity was significant (χ^2^(6) = 284.394, *p* < 0.001), indicating that the data were suitable for dimension reduction. PCA yielded a single-component solution with an eigenvalue of 3.832, accounting for 95.806% of the total variance. Component loadings were 0.989 for DHEAS, 0.970 for ALLO, 0.978 for testosterone, and 0.978 for E2.

The model was significant, R = 0.567, R^2^ = 0.322, adjusted R^2^ = 0.265, F(3, 36) = 5.693, *p* = 0.003. The hormone factor score (B = 2.390, *p* = 0.016) was a significant predictor, whereas API-EC (B = 0.166, *p* = 0.094) and DERS-16 were not (B = 0.117, *p* = 0.130); diagnostics indicated low multicollinearity (VIFs = 1.005–1.114) and Durbin–Watson = 1.916).

Hierarchical regression models further evaluated hormone-specific effects by entering API-EC and DERS-16 in Step 1 (F = 4.691, *p* = 0.015; adjusted R^2^ = 0.159) and adding each hormone separately in Step 2. DHEAS (F = 5.990, *p* = 0.002; adjusted R^2^ = 0.277; B = 7.150, *p* = 0.012), testosterone (F = 6.409, *p* = 0.001; adjusted R^2^ = 0.294; B = 8.282, *p* = 0.007), oxytocin (F = 5.383, *p* = 0.004; adjusted R^2^ = 0.252; B = 6.838, *p* = 0.023), and E2 (F = 5.412, *p* = 0.004; adjusted R^2^ = 0.253; B = 6.735, *p* = 0.023) were significant positive predictors, whereas ALLO was at trend level (B = 7.430, *p* = 0.055). After Benjamini–Hochberg FDR correction across the five hormone models, DHEAS, testosterone, oxytocin, and E2 remained significant (q < 0.05), whereas ALLO did not (Table 3).

## 4. Discussion

The present study investigated peripheral neuroactive steroids (ALLO, DHEAS, testosterone, E2) and oxytocin together with key psychosocial domains in men with methamphetamine use disorder (MUD) undergoing inpatient treatment. Patients showed substantially greater emotion dysregulation, insecure attachment (anxious and avoidant), aggression, and suicidal ideation than controls, whereas serum hormone and oxytocin levels did not differ between groups. Psychosocial measures clustered strongly with one another, while endocrine markers were highly intercorrelated, indicating considerable shared variance. In patients, craving severity was best explained by endocrine indices (composite and hormone-specific), whereas emotion dysregulation and addiction severity did not contribute in multivariable models. Finally, hormone-by-group interaction effects were not supported for DERS-16 or trait anger, despite robust adjusted group differences in these outcomes.

Serum levels of ALLO, DHEAS, testosterone, E2, and oxytocin did not differ statistically between patient and control groups in our study. While absolute hormone levels did not differ, the overall pattern of hormone–clinical associations in the total sample was limited in magnitude, with most correlations being small and non-significant. Notably, endocrine markers exhibited substantial shared variance, characterized by high intercorrelations across both the total sample and its subsamples. This indicates that these measures are likely capturing a common endocrine signal rather than reflecting independent hormonal effects. In this context, exploration of within-group patterns—such as the inverse associations observed between ALLO/testosterone levels and difficulties in emotion regulation in the control group—should be interpreted with caution. This is particularly important because formal moderation tests did not support interactions between group and hormone levels regarding emotion regulation or anger. Overall, these findings suggest that such links may be less detectable in this sample of hospitalized men with MUD under the present measurement conditions, which could reflect chronic neuroadaptations related to substance use [45,46]. In previous research involving patients diagnosed with bipolar mood disorder, serum levels of allopregnanolone (ALLO) were found to be significantly lower in individuals with marijuana addiction. However, no significant differences were observed in those with cocaine, amphetamine, or alcohol use disorders [28]. Additionally, it has been reported that serum levels of dehydroepiandrosterone (DHEA) and dehydroepiandrosterone sulfate (DHEAS) change in cocaine users following withdrawal; however, these serum levels were not influenced by cocaine-related factors such as duration, frequency, or amount of use [29]. The existing literature on the relationship between serum levels of neuroactive steroids (NAS) and substance use is limited, and our findings provide valuable insights into this emerging field of research.

There is significant evidence in the literature indicating that individuals with substance use disorders have more difficulty regulating emotions than individuals without substance use disorders [47,48]. In our study, the patient group reported significantly greater difficulties in emotion regulation compared to controls without substance use disorder, which aligns with previous findings. This finding suggests that men with MUD undergoing inpatient treatment experience greater emotion regulation difficulties than non-substance-use-disorder controls, possibly because individuals with preexisting difficulties in emotional regulation may turn to methamphetamine as a form of self-medication for emotional distress. Alternatively, chronic methamphetamine use itself may negatively affect individuals’ emotion regulation mechanisms [11].

Our patient group had significantly higher scores on both anxious and avoidant attachment subscales of the ECR-R compared to controls without substance use disorder, indicating more insecure attachment in adult relationships. There is significant evidence in the literature supporting a relationship between insecure attachment and substance use [49,50]. In a study conducted among adoptees, both anxious and avoidant attachment styles were found to be associated with substance use disorder, with no significant differences between attachment subtypes [51]. Consistent with previous literature, our study demonstrates that hospitalized men with MUD exhibit greater insecure attachment compared to controls without substance use disorder.

In previous research, serum cortisol and DHEAS levels were measured during the withdrawal period in male patients with cocaine addiction, and a gradual decrease in cortisol with a reciprocal increase in DHEAS levels was observed between the 6th and 18th days of abstinence [29]. In our current study, craving scores in patients with MUD were positively associated with serum DHEAS in bivariate analyses, and DHEAS also emerged as a significant positive predictor of craving in hormone-specific regression models after controlling addiction severity and emotion regulation, remaining significant after FDR correction. It is known that individuals with MUD exhibit neuroadaptations within the dopamine pathways [52]. Thus, elevated DHEAS levels may reflect a compensatory response to dopamine deficiency in individuals experiencing high levels of craving severity in this inpatient male MUD sample [53]. This interpretation is supported by preclinical findings indicating that DHEAS stimulates dopamine release within the central nervous system [54].

We also found positive correlations between craving scores and serum levels of testosterone and E2. Previous studies investigating E2 and testosterone in substance use disorders have primarily focused on gender differences [55,56,57,58]. However, the neuroactive steroid properties of testosterone and E2 are also recognized [59]. A recent study reported a significant positive correlation between serum testosterone levels and opioid craving [60]. In our study, testosterone and E2 were significant positive predictors of craving in hormone-specific regression models (controlling addiction severity and emotion regulation) and remained significant after FDR correction, indicating that these associations were not limited to nominal bivariate correlations. Nevertheless, the effect sizes were modest, and endocrine measures were highly intercorrelated, suggesting that testosterone and E2 may partly index a broader endocrine factor linked to craving intensity rather than fully independent effects.

Oxytocin levels did not differ between groups; however, oxytocin showed a positive association with craving in patients and emerged as a significant predictor in hormone-specific regression models, remaining significant after FDR correction. Given the context-sensitive and pulsatile nature of oxytocin, single-time-point serum levels may be more informative as state-linked correlates of craving during inpatient treatment than as markers of group differences. Future research should assess oxytocin dynamics with repeated sampling and designs that capture relevant contextual variation.

Regarding emotion regulation and anger outcomes, no group × hormone interactions were detected in ANCOVA moderation models, indicating that the associations of ALLO or testosterone with DERS-16 (and testosterone with trait anger) did not significantly differ between patients and controls. Importantly, group effects remained robust: patients had significantly higher DERS-16 and anger scores than controls after adjustment. In the fully adjusted anger models, testosterone showed an inverse association with trait anger (supported by bootstrap-confirmed regression), consistent with a potential regulatory link; however, the lack of interaction effects suggests that this association should not be interpreted as group-specific.

In the patient group, craving was more strongly associated with endocrine indices than with the other predictors included in the models: a hormone composite factor score significantly predicted craving, whereas emotion regulation and API-EC did not. In hormone-specific models, DHEAS, testosterone, oxytocin, and E2 were significant positive predictors and remained significant after FDR correction, whereas ALLO did not. Taken together, the results indicate that emotion dysregulation—despite being higher in patients and showing a modest bivariate correlation with craving—was attenuated in multivariable models, whereas endocrine indices, including a coordinated hormone profile (neurosteroids) captured by the composite factor score, remained significant predictors of craving during inpatient treatment, consistent with prior work linking craving to stress- and cue-related neuroendocrine mechanisms [61,62].

Conversely, in the broader sample, hormone–psychosocial correlations were generally small, with DHEAS showing modest inverse correlations with anger and emotional clarity. Exploratory within-group associations in controls (e.g., inverse links of ALLO and testosterone with DERS-16) should be interpreted cautiously because Group × hormone interactions were not supported in moderation models.

This study has several limitations. Firstly, it remains uncertain whether serum levels of neuroactive steroids (NAS) and oxytocin accurately reflect their levels in the central nervous system. Secondly, measuring hormone levels at only one time point (day 7) restricted our ability to assess the acute effects of methamphetamine and observe changes over the course of treatment. Additionally, the universal presence of tobacco use among participants made it difficult to control the potential confounding effects of smoking on hormone levels. Furthermore, the lack of precise dosing information for methamphetamine limited our ability to interpret the findings. In addition, the fact that the patient group consisted only of hospitalized men limits the generalizability of the findings to women, outpatients, and broader populations with methamphetamine use disorder. Moreover, cognitive impairments potentially associated with methamphetamine use were not assessed in this study, which may represent an additional limitation in interpreting the other measured parameters, since such deficits may be related to psychological and biological outcomes. Finally, excluding patients who dropped out or did not meet the inclusion criteria may affect the generalizability of our results.

Despite these limitations, this study has notable strengths. Both the patient and control groups underwent structured clinical evaluations using SCID-5-CV and SCID-5-PD interviews, which provided comprehensive psychiatric assessments. Furthermore, evaluating multiple clinical variables simultaneously allowed for a broader understanding of the relationships between hormones and behavior. Conducting the study in an inpatient clinic setting with an exclusively male sample created a homogeneous group, thereby reducing potential confounding factors, although it also limits generalizability. Lastly, confirming that all patients tested negative for methamphetamine in urine samples was vital for excluding the acute toxic effects of recent substance use.

## 5. Conclusions

In this inpatient study of men with methamphetamine use disorder, participants exhibited significantly greater impairments in affective and interpersonal functioning, along with higher rates of aggression and suicidal outcomes compared to the control group. Although there were no differences in peripheral neuroactive steroids and oxytocin levels between groups, the endocrine markers were strongly intercorrelated. Craving severity in patients was best explained by endocrine indices rather than by addiction severity and affective dysregulation. Group-by-hormone moderation effects were not supported for affective or anger outcomes, highlighting the complexity of the links between endocrine function and behavior, as well as the need for larger, longitudinal, hypothesis-driven studies with repeated hormonal assessments.

## Figures and Tables

**Table 1:** Comparison of sociodemographic and clinical data of the patient and control groups.

	Patient Group, *n* = 40	Control Group, *n* = 41	Statistics	*p*
Age mean ± SD	30.02 ± 6.52	32.26 ± 7.99	t = −1.382	0.171
Marital Status *n* (%)			χ^2^ = 5455	0.020
Married	17 (%42.5)	28 (%68.3)		
Single	23 (%57.5)	13 (%31.7)		
Place of residence *n* (%)			χ^2^ = 7.619	0.781
Urban	8 (20)	7 (17.1)		
Rural	32 (80)	34 (82.9)		
Working status *n* (%)			χ^2^ = 12.36	**0.001**
Working	17 (42.5)	33 (80.5)		
Not working	23 (57.5)	8 (19.5)		
Duration of breastfeeding in infancy (self-reported, months) ± SD	14.12 ± 8.77	15.02 ± 9.41		0.658
Education *n* (%)			χ^2^ = 16.37	**<0.001**
Primary school	2 (5)	1 (2.4)		
Middle-school	18 (45)	4 (9.8)		
High-school	16 (40)	34 (82.9)		
University	4 (10)	2 (4.9)		
BMI ± SD	24.16 ± 3.14	25.52 ± 2.25	t = −2.229	**0.029**
Smoking starting age ± SD	15.70 ± 4.69	17.09 ± 4.46	Z = −1.983	**0.047**
Frequency of use (methamphetamine) *n* (%)				
≥2 days per week	9 (22.5)			
6–7 days per week	31 (77.5)			
History of suicide *n* (%)			χ^2^ = 11.69	**<0.001**
There is history of suicide	10 (25)	0 (0)		
There is not history of suicide	30 (75)	41 (100)		
SCID-5-CV diagnosis *n* (%)				0.502
Major depressive disorder	2 (5)	3 (7.3)		
Anxiety disorder	1 (2.5)	1 (2.4)		
Panic Disorder	1 (2.5)	1 (2.4)		
Obsessive–compulsive disorder	1 (2.5)	0		
Adult ADHD	2 (5)	0		
SCID-5-PD diagnosis *n* (%)				**<0.001**
Paranoid personality disorder	1 (2.5)	0		
Borderline personality disorder	5 (12.5)	1 (2.4)		
Antisocial personality disorder	7 (17.5)	0		
Narcissistic personality disorder	2 (5)	1 (2.4)		
Dependent personality disorder	2 (5)	1 (2.4)		

Bold *p*-values indicate statistical significance (*p* < 0.05). SCID-5-CV/PD; Structured Clinical Interview for DSM-5-Disorders—Clinician Version/Personality Disorder.

**Table 2:** Comparison of the patient and control groups DERS-16, ECR-R, BPAQ and BSS.

	Patient Group, *n* = 40	Control Group, *n* = 41	*p*
DERS-16			
Clarity	5.9 ± 1.99	3.80 ± 1.70	**<0.001**
Impulse	8.02 ± 3.07	5.0 ± 2.25	**<0.001**
Aim	9.47 ± 3.04	7.07 ± 2.54	**<0.001**
Strategies	12.67 ± 5.08	8.02 ± 2.64	**<0.001**
Non-acceptance	8.17 ± 3.28	4.82 ± 2.15	**<0.001**
Total	44.25 ± 13.47	28.73 ± 8.16	**<0.001**
ECR-R			
Anxious Attachment	3.82 ± 1.19	3.04 ± 0.80	**0.001**
Avoidant Attachment	3.19 ± 1.13	2.47 ± 0.88	**0.002**
BPAQ	59.90 ± 18.08	33.90 ± 14.15	**<0.001**
BSS	6.65 ± 5.98	3.12 ± 4.36	**0.003**

Bold *p*-values indicate statistical significance (*p* < 0.05). DERS-16, Difficulties in Emotion Regulation Scale-16; ECR-R, Experiences in Close Relationships–Revised; BPAQ, Buss–Perry Aggression Questionnaire; BSS, Beck Scale for Suicide Ideation.

**Table 3:** Hierarchical regression (Enter) predicting craving.

Hormone	ΔR^2^	*p* (ΔR^2^)	Full Model F	*p*	AdR^2^	Hormone B	*p* (B)	Durbin–Watson	Max VIF	BH-FDR q
DHEAS_	0.131	0.012	5.990	0.002	0.277	7.150	0.012	1.918	1.116	0.0288
Testosterone	0.146	0.007	6.409	0.001	0.294	8.282	0.007	1.887	1.122	0.0288
Oxytocin_	0.107	0.023	5.383	0.004	0.252	6.838	0.023	1.840	1.106	0.0288
E2	0.109	0.023	5.412	0.004	0.253	6.735	0.023	1.915	1.115	0.0288
ALLO	0.079	0.055	4.692	0.007	0.221	7.430	0.055	1.893	1.105	0.0550

Block 1 (covariates-only): API-EC + DERS-16 total → F = 4.691, *p* = 0.015; adjusted R^2^ = 0.159, Block 2 single-hormone models (log-transformed).

## Data Availability

The original contributions presented in this study are included in the article/Supplementary Materials. Further inquiries can be directed to the corresponding author.

## Supplementary Materials

The following supporting information can be downloaded at: https://www.mdpi.com/article/10.3390/medsci14020169/s1, Table S1. Correlations Among Measures of Emotion Regulation Difficulties (DERS), Aggression (BPAQ), Attachment (ECR-R), and Suicidal Ideation; Table S2. Correlations of Age, Breastfeeding Duration, and BMI with Total Scores on the Main Scales; Table S3. Correlations Among Hormones in the Total Sample; Table S4. Correlations of hormones with clinical variables and scales; Table S5. Correlation Matrix in the Patient Subsample; Table S6. Correlation matrix in the control group.
