# Supplementary material for: The Relationship Between Serum Neurosteroids and Oxytocin Levels and Craving, Aggression and Emotion Regulation in Patients with Methamphetamine Use Disorder
Source: Med Sci (Basel). 2026 Mar 27;14(2):169. doi: 10.3390/medsci14020169 (PMC13108118; doi:10.3390/medsci14020169)
Supplement: Supplementary file 1 [file medsci-14-00169-s001.zip › medsci-4207638-supplementary.pdf]

Table S1. Correlations among emotion regulation difficulties (DERS), aggression (BPAQ), attachment (ECR-R), and suicide ideation

	Measure	1	2	3	4	5	6	7	8	9	10	11	12	13
		r(p)*	r(p)	r(p)	r(p)	r(p)	r(p)	r(p)	r(p)	r(p)	r(p)	r(p)	r(p)	r(p)
1	DERS-16 Total	—												
2	Clarity	0.738 (p<.001)	—											
3	Goals	0.759 (p<.001)	0.462 (p<.001)	—										
4	Impulse	0.858 (p<.001)	0.555 (p<.001)	0.608 (p<.001)	—									
5	Strategies	0.928 (p<.001)	0.605 (p<.001)	0.612 (p<.001)	0.760 (p<.001)	—								
6	Nonacceptance	0.835 (p<.001)	0.600 (p<.001)	0.474 (p<.001)	0.609 (p<.001)	0.748 (p<.001)	—							
7	BPAQ Total	0.668 (p<.001)	0.551 (p<.001)	0.437 (p<.001)	0.608 (p<.001)	0.594 (p<.001)	0.590 (p<.001)	—						
8	Physical Aggression	0.531 (p<.001)	0.511 (p<.001)	0.375 (p=.001)	0.551 (p<.001)	0.445 (p<.001)	0.369 (p=.001)	0.836 (p<.001)	—					
9	Verbal Aggression	0.410 (p<.001)	0.387 (p<.001)	0.272 (p=.014)	0.293 (p=.008)	0.385 (p<.001)	0.372 (p=.001)	0.744 (p<.001)	0.555 (p<.001)	—				
10	Anger	0.668 (p<.001)	0.523 (p<.001)	0.414 (p<.001)	0.618 (p<.001)	0.609 (p<.001)	0.599 (p<.001)	0.859 (p<.001)	0.591 (p<.001)	0.563 (p<.001)	—			
11	Hostility	0.529 (p<.001)	0.361 (p=.001)	0.339 (p=.002)	0.451 (p<.001)	0.469 (p<.001)	0.556 (p<.001)	0.820 (p<.001)	0.550 (p<.001)	0.487 (p<.001)	0.603 (p<.001)	—		
12	ECR-R Anxiety	0.547 (p<.001)	0.502 (p<.001)	0.397 (p<.001)	0.360 (p=.001)	0.452 (p<.001)	0.596 (p<.001)	0.504 (p<.001)	0.308 (p=.005)	0.312 (p=.005)	0.414 (p<.001)	0.591 (p<.001)	—	
13	ECR-R Avoidance	0.408 (p<.001)	0.468 (p<.001)	0.262 (p=.018)	0.289 (p=.009)	0.297 (p=.007)	0.449 (p<.001)	0.425 (p<.001)	0.320 (p=.004)	0.279 (p=.012)	0.394 (p<.001)	0.379 (p<.001)	0.464 (p<.001)	—
14	BSS	0.311 (p=.005)	0.227 (p=.041)	0.155 (p=.167)	0.162 (p=.148)	0.330 (p=.003)	0.379 (p<.001)	0.350 (p=.001)	0.198 (p=.076)	0.284 (p=.010)	0.346 (p=.002)	0.324 (p=.003)	0.219 (p=.049)	0.243 (p=.029)

(BSS)(N = 81) *Values are Pearson's r (two-tailed p). SPSS ".000" is reported as **p < .001**.

Table S2. Pearson correlations of age, breastfeeding duration, and BMI with main scale total scores (N = 81)

Scale	Age, *r* (*p*)	Breastfeeding* *r* (*p*)	BMI, *r* (*p*)
**DERS-16 Total**	−0.096 (.394)	−0.039 (.730)	−0.147 (.189)
Clarity	−0.138 (.220)	−0.145 (.196)	−0.177 (.114)
Goals	−0.096 (.394)	0.114 (.313)	−0.149 (.184)
Impulse	−0.132 (.241)	0.006 (.956)	−0.043 (.706)
Strategies	−0.050 (.656)	−0.111 (.322)	−0.101 (.367)
Nonacceptance	−0.023 (.836)	−0.020 (.860)	−0.174 (.119)
**BPAQ Total**	−0.075 (.506)	−0.003 (.979)	−0.144 (.198)
Physical Aggression	−0.130 (.248)	0.031 (.786)	−0.198 (.076)
Verbal Aggression	−0.085 (.452)	0.024 (.833)	−0.184 (.101)
Anger	0.087 (.442)	−0.058 (.606)	−0.042 (.710)
Hostility	−0.185 (.099)	−0.025 (.824)	−0.078 (.487)
**ECR-R Anxiety**	−0.021 (.852)	0.006 (.958)	**−0.243* (.029)**
**ECR-R Avoidance**	−0.146 (.193)	0.055 (.623)	−0.192 (.086)
**BSS**	0.071 (.529)	−0.154 (.170)	−0.167 (.136)

*Breastfeeding duration (months)

Table S3. Pearson correlations among hormones in Total Sample (n = 81)

Variable	1. DHEAS	2. Oxytocin	3. ALLO	4. Testosterone	5. Estrogen
**1. DHEAS**	—	0.933 (p<.001)	0.886 (p<.001)	0.936 (p<.001)	0.914 (p<.001)
**2. Oxytocin**		—	0.921 (p<.001)	0.906 (p<.001)	0.934 (p<.001)
**3. ALLO**			—	0.877 (p<.001)	0.908 (p<.001)
**4. Testosterone**				—	0.903 (p<.001)
**5. Estrogen**					—

Table S4. Pearson correlations of log-transformed hormones with clinical variables and scales (N = 81)

Variable	DHEAS	Oxytocin	ALLO	Testosterone	Estrogen
	***r* (*p*)**	***r* (*p*)**	***r* (*p*)**	***r* (*p*)**	***r* (*p*)**
Age	−0.103 (.358)	−0.134 (.234)	−0.206 (.064)	−0.139 (.215)	−0.114 (.310)
Smoking initiation age	0.030 (.789)	0.009 (.937)	−0.061 (.591)	0.004 (.971)	−0.020 (.859)
BMI	−0.065 (.567)	−0.101 (.371)	−0.109 (.331)	−0.125 (.268)	−0.115 (.308)
**DERS-16 Total**	−0.164 (.143)	−0.071 (.531)	−0.085 (.448)	−0.059 (.599)	−0.067 (.550)
DERS Clarity	−0.221 (.047)	−0.145 (.198)	−0.105 (.353)	−0.137 (.221)	−0.149 (.185)
DERS Goals	−0.209 (.061)	−0.159 (.156)	−0.157 (.162)	−0.107 (.344)	−0.152 (.175)
DERS Impulse	−0.131 (.245)	−0.075 (.504)	−0.060 (.593)	−0.041 (.715)	−0.029 (.795)
DERS Strategies	−0.064 (.570)	0.024 (.834)	−0.011 (.925)	0.011 (.921)	0.018 (.872)
DERS Nonacceptance	−0.128 (.257)	−0.013 (.906)	−0.069 (.540)	−0.034 (.762)	−0.040 (.726)
**BPAQ Total**	−0.195 (.081)	−0.063 (.574)	−0.084 (.458)	−0.098 (.383)	−0.047 (.679)
BPAQ Physical Aggression	−0.202 (.070)	−0.061 (.587)	−0.047 (.678)	−0.096 (.395)	−0.043 (.706)
BPAQ Verbal Aggression	0.020 (.857)	0.093 (.406)	0.124 (.271)	0.131 (.243)	0.093 (.410)
BPAQ Anger	**−0.258 (.020)**	−0.167 (.136)	−0.183 (.101)	−0.172 (.125)	−0.156 (.164)
BPAQ Hostility	−0.124 (.270)	−0.010 (.930)	−0.088 (.432)	−0.099 (.378)	0.010 (.927)
**ECR-R Anxiety**	−0.082 (.468)	0.015 (.894)	−0.016 (.884)	−0.012 (.916)	0.043 (.703)
**ECR-R Avoidance**	−0.080 (.478)	0.021 (.849)	−0.045 (.690)	−0.052 (.644)	−0.051 (.651)
**BSS Total**	0.019 (.869)	0.106 (.347)	0.061 (.586)	0.031 (.780)	0.051 (.654)

Values are Pearson's r with two-tailed p values in parentheses. DERS = Difficulties in Emotion Regulation Scale; BPAQ = Buss–Perry Aggression Questionnaire; ECR-R = Experiences in Close Relationships–Revised; BSS = Beck Scale for Suicide Ideation.

Table S5. Pearson correlation matrix in the patient subsample (N = 40)*

#	**Variable**	**1**	**2**	**3**	**4**	**5**	**6**	**7**	**8**	**9**	**10**	**11**	**12**	**13**	**14**
1	DHEAS	—													
2	Oxytocin	0.965 (p<.001)	—												
3	ALLO (AP_log)	0.944 (p<.001)	0.952 (p<.001)	—											
4	Testosterone	0.972 (p<.001)	0.930 (p<.001)	0.917 (p<.001)	—										
5	Estrogen (E2)	0.957 (p<.001)	0.948 (p<.001)	0.936 (p<.001)	0.938 (p<.001)	—									
6	**Age**	−0.467 (p=.002)	−0.493 (p=.001)	−0.523 (p=.001)	−0.438 (p=.005)	−0.470 (p=.002)	—								
7	API Total**	0.156 (p=.338)	0.108 (p=.507)	0.063 (p=.699)	0.201 (p=.214)	0.176 (p=.278)	0.017 (p=.918)	—							
8	DERS-16	−0.011 (p=.945)	0.029 (p=.859)	−0.022 (p=.891)	0.005 (p=.976)	0.013 (p=.938)	0.051 (p=.753)	0.409 (p=.009)	—						
9	BPAQ	−0.113 (p=.487)	−0.082 (p=.616)	−0.141 (p=.385)	−0.087 (p=.593)	−0.066 (p=.686)	0.085 (p=.601)	0.232 (p=.150)	0.549 (p<.001)	—					
10	Craving	0.384 (p=.014)	0.350 (p=.027)	0.284 (p=.076)	0.415 (p=.008)	0.360 (p=.023)	−0.056 (p=.733)	0.465 (p=.003)	0.359 (p=.023)	0.221 (p=.171)	—				
11	BMI	−0.268 (p=.094)	−0.275 (p=.085)	−0.291 (p=.068)	−0.330 (p=.037)	−0.271 (p=.091)	0.514 (p=.001)	−0.100 (p=.538)	−0.013 (p=.934)	0.166 (p=.306)	−0.263 (p=.101)	—			

12	Age at substance-use onset	−0.154 (p=.343)	−0.149 (p=.359)	−0.194 (p=.229)	−0.150 (p=.355)	−0.113 (p=.488)	0.556 (p<.001)	−0.103 (p=.526)	0.134 (p=.408)	0.130 (p=.426)	−0.035 (p=.829)	0.303 (p=.057)	—		
13	ECR-R Avoidant	0.071 (p=.665)	0.138 (p=.397)	0.099 (p=.544)	0.062 (p=.706)	0.049 (p=.762)	−0.238 (p=.138)	−0.152 (p=.349)	0.121 (p=.456)	0.169 (p=.296)	0.034 (p=.837)	−0.170 (p=.294)	−0.244 (p=.129)	—	
14	ECR-R Anxious	0.029 (p=.860)	0.076 (p=.639)	0.081 (p=.619)	0.076 (p=.642)	0.097 (p=.551)	−0.019 (p=.907)	0.067 (p=.683)	0.434 (p=.005)	0.323 (p=.042)	0.070 (p=.670)	−0.222 (p=.169)	0.305 (p=.055)	0.266 (p=.097)	—
15	**BSS Total**	−0.047 (p=.772)	0.026 (p=.876)	−0.029 (p=.860)	−0.081 (p=.621)	−0.077 (p=.638)	0.146 (p=.369)	0.002 (p=.988)	0.217 (p=.179)	0.201 (p=.214)	0.277 (p=.084)	−0.043 (p=.791)	−0.096 (p=.555)	0.199 (p=.219)	0.182 (p=.262)

**Hormones are log-transformed, ** Correlation with carving score and* API Total excluded craving subscale score; r= 0.367 p=.020,

Table S6. Pearson correlation matrix in the control group (*N* = 41).

#	Variable	1	2	3	4	5	6	7	8	9	10	11	12
1	DHEAS	—											
2	Oxytocin	0.916 (p<.001)	—										
3	ALLO	0.855 (p<.001)	0.896 (p<.001)	—									
4	Testosterone	0.933 (p<.001)	0.888 (p<.001)	0.841 (p<.001)	—								
5	Estrogen	0.885 (p<.001)	0.922 (p<.001)	0.885 (p<.001)	0.875 (p<.001)	—							
6	Age	0.136 (p=.395)	0.128 (p=.424)	0.044 (p=.785)	0.099 (p=.537)	0.157 (p=.328)	—						
7	DERS-16 Total	−0.293 (p=.063)	−0.264 (p=.095)	−0.320 (p=.042)	−0.310 (p=.048)	−0.222 (p=.163)	−0.090 (p=.577)	—					
8	BPAQ Total	−0.211 (p=.185)	−0.080 (p=.617)	−0.144 (p=.369)	−0.285 (p=.071)	−0.038 (p=.813)	−0.028 (p=.864)	0.348 (p=.026)	—				
9	BMI	0.122 (p=.448)	0.107 (p=.506)	0.143 (p=.372)	0.161 (p=.313)	0.074 (p=.644)	−0.003 (p=.984)	0.004 (p=.982)	−0.254 (p=.109)	—			
10	ECR-R Avoidant	−0.179 (p=.263)	−0.108 (p=.502)	−0.260 (p=.101)	−0.244 (p=.125)	−0.174 (p=.276)	0.034 (p=.834)	0.599 (p<.001)	0.481 (p=.001)	−0.032 (p=.842)	—		
11	ECR-R Anxious	−0.143 (p=.373)	−0.062 (p=.700)	−0.197 (p=.218)	−0.192 (p=.230)	−0.015 (p=.926)	0.108 (p=.503)	0.474 (p=.002)	0.494 (p=.001)	−0.069 (p=.667)	0.621 (p<.001)	—	
12	**BSS Total**	0.201 (p=.209)	0.225 (p=.157)	0.156 (p=.329)	0.132 (p=.412)	0.232 (p=.144)	0.119 (p=.457)	0.041 (p=.799)	0.198 (p=.215)	−0.194 (p=.224)	0.065 (p=.689)	−0.014 (p=.933)	—

Cells show r *(p). Hormones are log-transformed*
